# Early Detection of Sudden Cardiac Death by Using Ensemble Empirical Mode Decomposition-Based Entropy and Classical Linear Features From Heart Rate Variability Signals

**DOI:** 10.3389/fphys.2020.00118

**Published:** 2020-02-25

**Authors:** Manhong Shi, Hongxin He, Wanchen Geng, Rongrong Wu, Chaoying Zhan, Yanwen Jin, Fei Zhu, Shumin Ren, Bairong Shen

**Affiliations:** ^1^Center for Systems Biology, Soochow University, Suzhou, China; ^2^College of Information and Network Engineering, Anhui Science and Technology University, Fengyang, China; ^3^Applied Mathematical Sciences, University of Connecticut, Storrs, CT, United States; ^4^School of Computer Science & Technology, Soochow University, Suzhou, China; ^5^Institutes for Systems Genetics, West China Hospital, Sichuan University, Chengdu, China

**Keywords:** sudden cardiac death, heart rate variability (HRV), ensemble empirical mode decomposition (EEMD), entropy, classical linear features

## Abstract

Sudden cardiac death (SCD), which can deprive a person of life within minutes, is a destructive heart abnormality. Thus, providing early warning information for patients at risk of SCD, especially those outside hospitals, is essential. In this study, we investigated the performances of ensemble empirical mode decomposition (EEMD)-based entropy features on SCD identification. EEMD-based entropy features were obtained by using the following technology: (1) EEMD was performed on HRV beats to decompose them into intrinsic mode functions (IMFs), (2) five entropy parameters, namely Rényi entropy (RenEn), fuzzy entropy (FuEn), dispersion Entropy (DisEn), improved multiscale permutation entropy (IMPE), and Renyi distribution entropy(RdisEn), were computed from the first four IMFs obtained, which were named EEMD-based entropy features. Additionally, an automated scheme combining EEMD-based entropy and classical linear (time and frequency domains) features was proposed with the intention of detecting SCD early by analyzing 14 min (at seven successive intervals of 2 min) heart rate variability (HRV) in signals from a normal population and subjects at risk of SCD. Firstly, EEMD-based entropy and classical linear measurements were extracted from HRV beats, and then the integrated measurements were ranked by various methodologies, i.e., *t*-test, entropy, receiver-operating characteristics (ROC), Wilcoxon, and Bhattacharyya. Finally, these ranked features were fed into a k-Nearest Neighbor algorithm for classification. Compared with several state-of-the-art methods, the proposed scheme firstly predicted subjects at risk of SCD up to 14 min earlier with an accuracy of 96.1%, a sensitivity of 97.5%, and a specificity of 94.4% 14 min before SCD onset. The simulation results exhibited that EEMD-based entropy estimators showed significant difference between SCD patients and normal individuals and outperformed the classical linear estimators in SCD detection, the EEMD-based FuEn and IMPE indexes were particularly useful assessments for identification of patients at risk of SCD and can be used as novel indices to reveal the disorders of rhythm variations of the autonomic nervous system when affected by SCD.

## Introduction

Sudden cardiac death (SCD) describes the death of a person who has died from previously known or even unknown cardiac diseases in an unanticipated and abrupt manner, within no more than an hour after the first occurrence of symptoms (Zipes and Wellens, 1998; Chugh, 2001; Myerburg and Castellanos, 2005). Nearly 300,000 lives in the United States and 700,000 lives in Europe are lost because of SCD each year (Lloyd-Jones et al., 2010; Soliman et al., 2011; Pagidipati and Gaziano, 2013). Approximately 5–37 out of 1000,000 young people die from SCD, and the occurrence rate of SCD in men is higher than that in women (Eckart et al., 2011; Sessa et al., 2018). Despite the increased usage of public defibrillation devices after collapse, according to the latest data, out-of-hospital survival is at only about 10.4% due to the failure to provide patients with timely care (Vandenberg et al., 2017). These startling figures highlight the significance of early SCD prediction for improving survival rates.

Most astonishingly, whether a person suffers from SCD has little to do with their history of heart disease, although most SCD subjects did have previously diagnosed or even undiagnosed cardiac abnormality (Jones and Tovar, 2002). Coronary thrombosis, causing blockages in the walls of blood vessels, is responsible for a significant number of SCD cases. The second leading cause of SCD is ventricular fibrillation (VF) in the adult population. VF is considered to be the potential mechanism in 20% of SCD episodes; this usually occurs before sudden cardiac arrest (SCA) and results in failure of the heart to pump blood, and unattended SCA subsequently leads to death (Pagidipati and Gaziano, 2013). The survival rates after VF decrease by about 10% per minute (Rea and Page, 2010). The Public Access Defibrillation (PAD) technique is usually used to rescue the dying after the collapse, but for patients outside hospitals it is difficult to provide timely and effective treatment in a short time, and therefore early detection of unanticipated SCD in a person suffering from VF is of vital significance for increasing the survival rate of out-of-hospital patients. Evaluation of electrocardiogram (ECG) and heart rate variability (HRV) signals has been regarded as a non-invasive tool for picking up minute differences among various classes to diagnose cardiovascular diseases. Along with the fast development of cloud computing and wearable sensors such as clothing, caps, watches, shoes, etc., this provides us with the chance to remotely monitor ECG/HRV signals of patients at high risk in a real-time, continuous manner (Hasan and Shahjalal, 2019; Steinberg et al., 2019; Toral and Garcia, 2019). Proposing the automated SCD prediction algorithm and combining the algorithm with an ECG real-time monitoring system is promising for providing early warning information so that the clinicians will have sufficient time to provide timely and effective treatment for patients at risk of SCD.

In previous studies with ECG and HRV, QT dispersion/interval, QRS duration, and signal-averaged ECG (SAECG) extracted from ECG signals by using linear methods were often used for predicting SCD (Viskin and Barron, 1997; Lombardi et al., 2001; Huikuri et al., 2003; Yeung et al., 2012; Bai et al., 2017). However, the assessment of QT interval showed negative results for its prognostic ability (Statters et al., 1994). HRV, obtained by computing the time of two successive R-waves within an ECG signal (Constant et al., 1999), has proven to be an independent indicator of mortality after MI (Malik, 1996). There are primarily three methods, namely the classical linear method (including time domain and frequency domain), time-frequency, and the non-linear method, that have been used for the analysis of HRV signals. A study reported (VanHoogenhuyze et al., 1989) that, compared to normal groups, the statistic values [e.g., standard deviation (SD) of the mean sinus R–R intervals (SDANN), mean of SD] obtained from HRV in SCD groups were lower. Apart from HRV signal analysis in the time domain mentioned above, Shen et al. (2007) applied fast Fourier transforms (FFT) to the HRV signals to acquire the frequency response. Various standard segments, known as high frequency (HF), low frequency (LF), and very low frequency (VLF), were found to be strong indicators of SCD (Shen et al., 2007). In the time-frequency domain, the Wigner–Ville transform, smoothed pseudo-Wigner–Ville distribution (SPWVD), and short-time Fourier transform were performed on the HRV signals to get corresponding time-frequency features for SCD prediction (Martinmäki et al., 2006; Ebrahimzadeh and Pooyan, 2011; Ebrahimzadeh et al., 2014; Mirhoseini et al., 2016). Some research has demonstrated that compared to the classic methods for HRV signal analysis, the non-linear methods, such as symbolic dynamic (Maestri et al., 2007), renormalized entropy (Voss, 1996), conditional entropy (Porta et al., 2000, 2001, 2017), and mutual non-linear prediction (Faes et al., 2008), were better able to find the complexities underlying the HRV signals due to the non-stationary and non-linear characteristics of these signals (Martis et al., 2012). In addition, the performance of non-linear features in distinguishing SCD subjects from normal in each 1-min HRV signal is more stable (Ebrahimzadeh et al., 2017).

Acharya et al. (2015a) proposed combined algorithms with non-linear features and wavelet transform, which were performed on HRV signals and showed an ability to predict SCD 4 min before its occurrence (Fujita et al., 2016). Nevertheless, the selection of a suitable basis function of wavelet transform for the signal analysis was not easy because the basis function was not adaptive to decomposed signals. Ensemble empirical mode decomposition (EEMD) and empirical mode decomposition (EMD) are adaptive signal decomposition methods that decompose the signals into intrinsic mode functions (IMFs) without prior knowledge and only according to the characteristics of the signal itself, which is vitally important for non-linear and non-stationary signal analysis (Wu and Huang, 2009). These signal decomposition methods have shown their capacity in various applications, such as the classification of ECG heartbeats (Rajesh and Dhuli, 2017), detection of shockable ventricular arrhythmia (Tripathy et al., 2016), and automated identification of congestive heart failure (Acharya et al., 2016).

**FIGURE 1:**
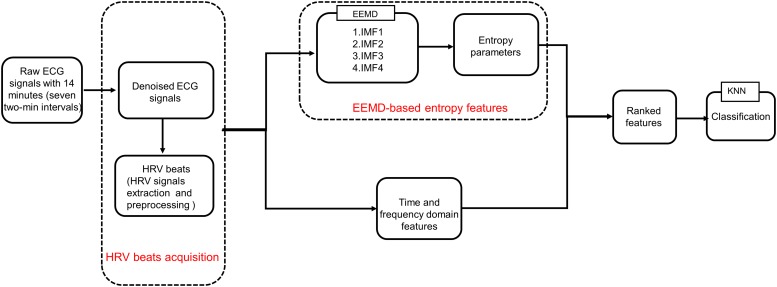
Proposed block diagram.

In the current study, we explored the performance of EEMD-based entropy metrics on SCD detection and proposed an automated SCD scheme based on EEMD and classical linear methods. Firstly, three time-domain, four frequency-domain, and 20 EEMD-based entropy features (five entropy indices were calculated from the first four IMFs obtained by EEMD) were extracted from HRV beats for early SCD identification. A block diagram of the proposed scheme is exhibited in Figure 1. Furthermore, the combination of classical linear and EEMD-based entropy features was ranked by various methods, i.e., *t*-test, entropy, receiver-operating characteristics (ROC), Wilcoxon, and Bhattacharyya. These ranked features were subjected to *k*-Nearest Neighbor (*k*-NN) classification to differentiate normal patients and those at risk of SCD.

## Materials and Methods

### Data Acquisition

In the current study, two databases, namely PhysioBank MIT-BIH Normal Sinus Rhythm (NSR) and MIT/BIH SCD, were employed to conduct a target assessment for the proposed method. The SCD database includes 23 24h-ECG recordings before SCD onset as well as a few seconds later. These patients, with a history of heart attack or hard tachyarrhythmia, were more likely to have SCD and to be affected eventually (Ebrahimzadeh et al., 2019). Details of the data used in this work are shown in Table 1. Of the 23 SCD subjects, only 20 patients (eight females, 10 males and two of unknown sex, aged 18–89) were used for further analysis in this work, because the ECG signals of the other three subjects did not show any VF episodes. A total of 36 ECG recordings from the MIT-BIH NSR and 40 SCD ECG signals were utilized from the SCD database. With the aim of maintaining consistent sampling between normal groups and subjects at risk of SCD, all the ECG signals used in this paper were resampled at 360 HZ.

**TABLE 1:** Details of the data used in this work.

**Database**	**Diagnosis**	**Number of subjects**	**Subjects features**	**Number of leads**	**Sampling rate**	**Number of ECG signals used**	**Length of record**
MIT-BIH NSR	Normal	18	13 females (age 20–50)	2	128HZ	36	14 min
			5 males (age 26–45)				
MIT-BIH SCD	SCD	23	8 females, 13 males, 2 sex unknown (age 18–89)	2	250HZ	40	14 min

### HRV Signal Extraction and Pre-Processing

**FIGURE 2:**
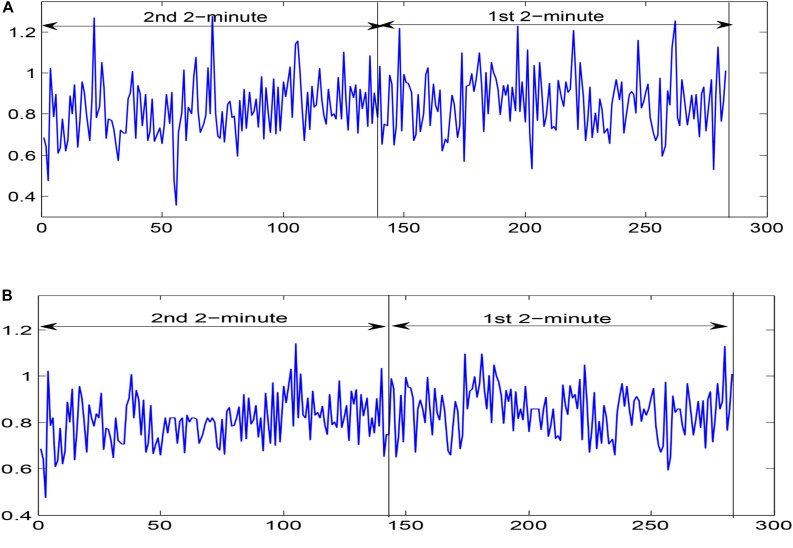
Two 2-minute **(A)** uncorrected HRV signals and **(B)** corrected HRV signals before SCD occurrence extracted from lead I of the ECG recording for patient number 35.

For 24 h of ECG recordings of the SCD patients, only ECG signals 14 min before VF onset were used to simulate 14 min before SCD. For the normal subjects, 14-min durations of ECG signal were chosen randomly. ECG signal collection contains interference from various noises, including baseline wander (<0.5 HZ) and power line interference (>50 Hz) (Zhao et al., 2013). The DWT with Daubechies order-6 wavelet basis method, which is applicable to non-stationary signals (Singh and Tiwari, 2006; Elhaj et al., 2016), was used in ECG signal denoising by setting the first two detail coefficients and the highest-level approximation coefficients to zero. Then, the denoised ECG signals were subjected to the Pan Tompkins algorithm, aiming at QRS complex detection (Pan and Tompkins, 1985), and, thereupon, corresponding HRV signals were determined. HRV signal pre-processing was essential before HRV signal analysis due to the fact that missed/false R peaks brought about ectopic intervals so as to generate poor quality HRV signals. In this paper, we removed unexpected data points in which RR intervals were more than 20% with respect to the median value of the next five and previous five RR intervals by adopting a median filter of five width method (Vest et al., 2018; Chen et al., 2015) for corrected HRV signal acquisition. In this work, the corrected HRV signals 14 min before SCD were uniformly divided into seven 2-min intervals (i.e., the 1st 2 min, 2nd 2 min, 3rd 2 min, etc.), and normal HRV signals of 14 min durations chosen randomly were similarly, partitioned into seven 2-min intervals. A 4-min uncorrected and corrected HRV signal before SCD occurrence (two 2-minute intervals), extracted from lead I of the ECG recording for patient number 35 are shown in Figure 2.

### Analysis of HRV Signals

#### Time and Frequency Domain Analysis

Frequently used estimators for time-domain analysis of HRV signals for SCD detection include the square root of the mean squared differences of adjacent normal-normal (NN) intervals (RMSSD), standard deviation of NN-intervals (SDNN), and proportion of NN-interval differences greater than 50 ms (pNN50). In frequency domain analysis of HRV signals, VLF, LF, HF, and LF/HF were calculated. The indices VLF, LF, and HF represent the spectral power in the very-low-frequency band (0.003–0.04), low-frequency band (0.04–0.15 Hz), and high-frequency band (0.15–0.4 Hz), respectively (Malik, 1996; Ebrahimzadeh et al., 2014).

#### Ensemble Empirical Mode Decomposition-Based Entropy Analysis

##### Empirical mode decomposition (EMD)

Empirical mode decomposition (EMD), introduced by Huang et al. (1998), is an adaptive signal decomposition mechanism without any prior criteria. A signal is decomposed into amplitude- and frequency-modulated (AM–FM) oscillatory components, termed IMFs, by using a sifting process. EMD is a greedy algorithm and has the ability to detect local information hidden in the signal. The process of EMD on a signal *x*(*t*) is illustrated as follows:

Step 1: Initialize *r*_0_ = *x*(*t*) and k = 1
Step 2: Compute the *k*^*th*^ IMF;
  (1) Initialize *b*_*k*_(*i*−1) = *r*_0_, where *i* = 1;
  (2) Extract all the local extremes (minima and maxima) of *b*_*k*_(*i*−1);
  (3) Interpolate the local minima and maxima based on a cubic spline function to obtain the corresponding lower and upper envelopes $(e_{m\,i\,n}^{k}$ and $e_{m\,a\,x}^{k}$);
  (4) Compute the mean: $m_{k}\,\left(i-1\right)=\left(e_{m\,i\,n}^{k}+e_{m\,a\,x}^{k}\right)/2$
  (5) Let *b*_*ki*_ = *b*_*k*_(*i*−1)−*m*_*k*_(*i*−1);
  (6) Compute $\text{D}=\sum_{t=0}^{n}{\left|\frac{b_{k}\,\left(i-1\right)-b_{k\,i}}{b_{k}\,\left(i-1\right)}\right|}^{2}$; when D is less than a previously set threshold, set *IMFk* = *b*_*ki*_; when *b*_*ki*_ is an IMF, n is the number of samples in total; otherwise go to step (2), where i = i + 1;
Step 3 Define *r*_*k* + 1_ = *r*_*k*_−*IMFk*;
Step 4 If *r*_*k+1*_ has at least two extrema, go to step 2, or else *r*_*k+1*_ is the residue

$$x\,\left(t\right)=\sum_{k=1}^{K}I\,M\,F\,k+r_{k+1}$$

##### EEMD

There existss a mode-mixing phenomenon, named an IMF, including different amplitude oscillations or parallel oscillations that reside in different IMFs. In the EMD method, due to the fact that the precondition of reasonable IMFs obtained from the EMD method is the occurrence of the extreme points and the distribution of the extreme points, the intermittency of IMFs will cause an appearance of mode mixing. To deal with the limitation of the EMD method (Wu and Huang, 2009), EEMD, an EMD signal decomposition method that is modified through adding white Gaussian noise with finite amplitude that evenly distributes the whole time-frequency space, was proposed. The various scale components of the original signal are mapped to suitable scales of reference built by the added white noise component. Although each decomposition trial results in noisy results because of the decomposed signals constituting the added white noise and the original signal, evenly distributed white noise is completely removed by computing the ensemble mean of all trials, while the original signal is preserved in the ultimate ensemble mean. Through this method, the mode-mixing phenomenon of EMD is effectively avoided (Zhao et al., 2013). The exact IMFs are given by the EEMD, and the calculation of EEMD is illustrated as follows:

Step 1 Add white Gaussian noises (**n^i^**(**t**),**i** = 0,⋯,**L**) with different SD to the signal **x**(**t**)

$${\text{x}}^{i}\,\left(t\right)=\text{x}\,\left(t\right)+{\text{n}}^{i}\,\left(t\right)$$

Step 2 Each ensemble signal **x^i^**(**t**) is subjected to EMD, with the aim of obtaining the IMFs ${\text{a}}_{k}^{i}\,\left(t\right)$ (**k** = 1,2,⋯,**K**).
Step 3 Calculate the **K^th^** IMF of the ensemble signal **x^i^**(**t**)

$$\bar{\text{IMFk}}=\frac{1}{\text{L}}\,\sum_{i=1}^{L}{\text{a}}_{k}^{i}\,\left(t\right)$$

**FIGURE 3:**
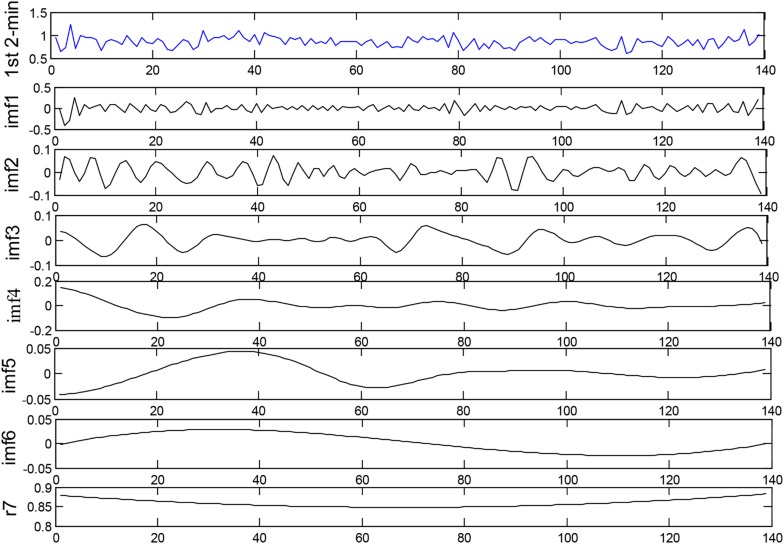
Decomposition of the 1st 2-min interval HRV signal before SCD occurrence by using the EEMD-based technique.

In the present work, EEMD (*D* = 0.2) is used to decompose each HRV signal segment of 2-min intervals into several IMFs for the subsequent extraction of corresponding features. We use the first four IMFs obtained from EEMD for SCD detection. Figure 3 depicts the decomposition of the 1st 2-min interval HRV signal before SCD occurrence, which was extracted from a SCD patient (number 35) by using the EEMD-based technique.

##### Entropy feature parameter

It is challenging to extract crucial features from HRV signals due to their non-linear and non-stationary characteristics. In this work, five entropy parameters, namely RenEn, FuEn, DisEn, RdisEn, and IMPE, were applied to the first four levels of IMFs obtained from EEMD for detecting abnormalities within prone-to-SCD HRV signals.

###### Rényi entropy

This parameter is capable of evaluating the spectral complexity in time series and is a generalized form of Shannon entropy (Faust and Bairy, 2012). The definition of RenEn follows as

$$R\,e\,n\,E\,n=\frac{1}{1-q}\,l\,o\,g_{2}\,\left(\sum_{i=1}^{n}p_{i}^{q}\right),q>0,q\neq 1$$

Fourier transformation is performed on HRV signals to acquire the power spectral density (PSD), and then the Fourier transforms of HRV signals are calculated, aiming at obtaining the power level of each frequency denoted by *p*_*i*_. *p*_*i*_ is computed by $\frac{P_{i}}{\sum P_{i}}$, where ∑*p*_*i*_ represents the total power. In this work, q = 2 was used, which is known as Rényi quadratic entropy and is widely applied in signal analysis (Kannathal et al., 2005; Sharma et al., 2015).

###### Fuzzy entropy

This entropy quantifies the complexity of a time series. Unlike SamEn with the Heaviside function, the similarity of vectors using this entropy is calculated on the basis of soft fuzzy membership functions. FuEn as a measurement of randomness that has less dependence on data length and stronger consistency and can achieve satisfying results in quantifying signals with various irregularities. Larger complexity within HRV signals results in a larger value of FuEn (Azami et al., 2017). Considering a time series *x*(*i*) of length *N*, we construct a-dimensional vectors $X_{i}=\left\{x\left(i\right),x\left(i+1\right),\cdots ,x\left(i+a-1\right)\right\}-a^{-1}\sum_{k=0}^{a-1}x\left(i+k\right),\left\{1\leq i\leq N-a+1\right\}$. The entropy is computed as

$$F\,u\,E\,n=-\ln\left(\phi^{a+1}\,/\,\phi^{a}\right)$$

where ϕ^a^ is calculated as

$$\phi^{a}=\frac{1}{N-a}\,\sum_{\text{i}=1}^{N-a}\left(\frac{1}{N-a-1}\,\sum_{j=1,j\neq i}^{N-a}D_{i\,j}^{a}\right),D_{i\,j}^{a}=\exp\left(-{\left(d_{i\,j}^{a}\right)}^{p}/\text{r}\right)$$

where FuzEn power p and tolerance r are given in advance, and the similarity degree $d_{i\,j}^{a}$ is the maximum distance between vectors _**Xi**_ and _**Xj**_(**i**≠**j**). Similarly, we compute ϕ^a+1^ for a + 1-dimensional vectors (Zhao et al., 2016). *a* = 2, *r* = 0.15 × SD (SD represents the standard deviation of a signal analyzed), and *p* = 2 were used in this work since it had been recommended by previous work (Li et al., 2015).

###### Dispersion entropy

Dispersion entropy (DisEn), a powerful and fast algorithm for measuring the randomness of signals, was introduced by Azami et al. (Rostaghi and Azami, 2016). It can simultaneously explore the amplitude and frequency variation of signals. For time series *x(i)* of length N, the DisEn index is computed as follows:

(1) The original time series *x(i)* of length N map into y from 0 to 1 by the normal cumulative distribution function as follows:

$$y_{i}=\frac{1}{\sigma \,\sqrt{2\,\pi }}\,\int_{-\infty }^{x_{i}}e^{\frac{-\left(t-\mu \right)}{2\,\sigma^{2}}}\,dt$$

where μ and σ represent the mean and standard deviation of time series.

(2) *y*_*i*_ is mapped to a group with integer indexes from 1 to *a*. To do this, *y*_*i*_ is multiplied by *a* and then summed with 0.5. Therewith, *y*_*i*_ is equal to its nearest integer according to the rounding method.

$$z_{i}^{a}=\text{round}\left(a.y_{i}+0.5\right)$$

where $z_{i}^{a}$ is the *i*th element of the group ^*za*^

(3) $z_{j}^{m,a}=\left\{z_{j}^{a},z_{j+d}^{a},\cdots ,z_{j+\left(m-1\right)\,d}^{a}\right\}$ are constituted by *m* (embedding dimension) and *d* (time delay)
(4) Each time series $z_{j}^{m,a}$ is mapped to a dispersion pattern π_*u_0 u_1…u_m–1*_

$$z_{i}^{a}=u_{0},z_{i+d}^{a}=u_{1},z_{i+2\,d}^{a}=u_{2},\cdots ,z_{i+\left(m-1\right)\,d}^{a}=u_{m-1}$$

(5) For each of ^*am*^ dispersion patterns π_*u_0 u_1…u_m–1*_, probability is computed by

$$p\,\left(\pi_{u_{0}\,u_{1}\,\cdots \,u_{m-1}}\right)$$

$$\;=\frac{N\,u\,m\,b\,e\,r\,\left\{j|j\leq N-\left(m-1\right)\,d,z_{j}^{m,a}\,h\,a\,s\,\pi_{u_{0}\,u_{1}\,\cdots \,u_{m-1}}\right\}}{N-\left(m-1\right)\,d}$$

(6) DisEn is calculated with m (embedding dimension) and a (number of groups) by

$$\text{DisEn}=-\sum_{\pi =1}^{a^{m}}p\,\left(\pi_{u_{0}\,u_{1}\,\cdots \,u_{m-1}}\right).\ln\left(p\,\left(\pi_{u_{0}\,u_{1}\,\cdots \,u_{m-1}}\right)\right)$$

*m* = 2, *s* = 6, and *d* = 1, recommended by reference (Rostaghi and Azami, 2016), were used in this work.

###### Rényi distribution entropy

Rényi distribution entropy (RdisEn), proposed in our previous work (Shi et al., 2019), is computed on the basis of the empirical probability distribution function (ePDF) of vector-to-vector distances from a given time series. Simulation results showed that parameter selection has little effect on the RdisEn measurement and that it has the reliable capacity to measure the complexity of short-term RR intervals data. For time series *x*(*i*) of length N, a-dimensional vectors *X*_*i*_ = {*x*(*i*),*x*(*i* + 1),⋯,*x*(*i* + *a*−1)}, {1≤*i*≤N-a}, are formed, and calculation of this entropy is defined as

$$R\,d\,i\,s\,E\,n=\frac{1}{\left(1-q\right)\,l\,o\,g_{2}^{\left(B\right)}}\,l\,o\,g_{2}\,\left(\sum_{t=1}^{B}p_{t}^{q}\right)$$

where *p*_*b*_,*b* = 1,2,⋯,*M* is the probability and is obtained using the following steps.

(1) Compute distance matrix D = {*d*_*ij*_}, where *d*_*ij*_ is the maximum distance between vectors *X*_*i*_ and *X*_*j*_{1≤*i*,*j*≤*N*−*a*}.
(2) Measure probability density by applying the histogram method to the distance matrix D, where M denotes bins of the histogram (Li et al., 2015). In this work, we use *a=2*, *M=512*, and 1.1 < *q* < 2.

###### Improved multiscale permutation entropy

Improved multiscale permutation entropy (IMPE), proposed by Azami and Escudero (2016), can quantify the dynamics of signals over multiple temporal scales, in contrast to the conventional entropy parameters such as sample entropy and permutation entropy, and it has superior reliability of entropy measurement for short-term time series. The IMP algorithm is performed as follows:

(1) Construct coarse-grained sequences $z_{i}^{\left(s\right)}=\left\{y_{i,1}^{\left(s\right)},y_{i,2}^{\left(s\right)},\cdots \right\}$, where $y_{i,j}^{\left(s\right)}=\frac{\sum_{f=0}^{s-1}x_{f+i+s\,\left(j-1\right)}}{s}$ for time series *x*(i) of length *N.*
(2) Compute permutation entropy of each $z_{i}^{\left(s\right)}$ for a s (scale factor)

$$\text{IMPE}=\frac{1}{s}\,\sum_{i=1}^{s}P\,E\,\left(z_{i}^{\left(s\right)},m\right)$$

where PE represents permutation entropy and m denotes embedding dimension. The details of the computing process of PE are described in Azami and Escudero (2016); *m* = 3 and *s* = 2,3,4,5,6 were used in this work.

RenEn is also called spectral entropy, as its calculation relates to the power spectrum. FuEn, DisEn, IMPE, and RdisEn are broadly classified as embedding entropies due to the fact that their calculations refer to the reconstruction of time series to measure the amount of randomness (Faust and Bairy, 2012). The five entropy parameters quantify the complexity and randomness within HRV signals derived from normal and SCD patients from different computational perspectives in the time series.

### Feature Assessment

Statistical analysis methods, including *t*-test and receiver-operating characteristics (ROC) analysis, were employed in this work, in order to determine the statistical significances and classification performances of features obtained. Where the *p*-value generated from *t*-test for a feature is less than 0.05, the feature is considered as of statistical significance, and the smaller the *p*-value is, the better the significance (Box, 1987). In ROC analysis, area under the curve (AUC) is used as an index to evaluate the classification ability of a feature. An AUC value is closer to 1 suggests better differentiation ability of the feature, whereas an AUC value closer to 0.5 implies worse separation ability.

### Feature Ranking

A total of 27 features (seven time-frequency domain and 20 EEMD-based entropy features) were obtained from the above steps, however, not all the features acquired are crucial for differentiating normal from SCD classes. Manual identification of features with significant contributions to SCD detection is extraordinarily tedious work. In this paper, four ranking methodologies, namely *t*-test, entropy, ROC, Wilcoxon, and Bhattacharyya, are utilized for ranking features. The *t*-test technology and ROC methods have been described in section “Feature Assessment.” In the entropy method, the features are ranked by relevance in descending order; the method is proposed based on the fact that lower irregularity corresponds with low entropy and vice versa. The Wilcoxon method evaluates the difference between the two correlative samples and is suitable for analyzing two different assessment sets derived from the same data. Bhattacharyya determines divergence between statistical populations by probability distributions; the features are ranked by their capacity to discriminate the training data (Acharya et al., 2015c).

### Classification

In order to separate the SCD and normal subjects, two types of k-NN (Mitchell, 1997) where *k* = 1,10, that is, 1-NN and 10-NN, were used in this study. With the aim of evaluating the performance of the two classifiers, three evaluators called accuracy, sensitivity, and specificity were calculated by using formulas introduced in Ebrahimzadeh et al. (2018b). Additionally, the 10-fold cross-validation method was employed. The dataset used in this method was randomly divided into 10 mutually exclusive parts with the same samples, where nine datasets were utilized for training and the remaining one was for testing. This calculation process was repeated 10 times. The three evaluators were computed for each calculation process. The average values of the three evaluators were obtained for the 10-times calculation process at the end, and we used these average values to assess the performance of the classifier used.

## Results

**FIGURE 4:**
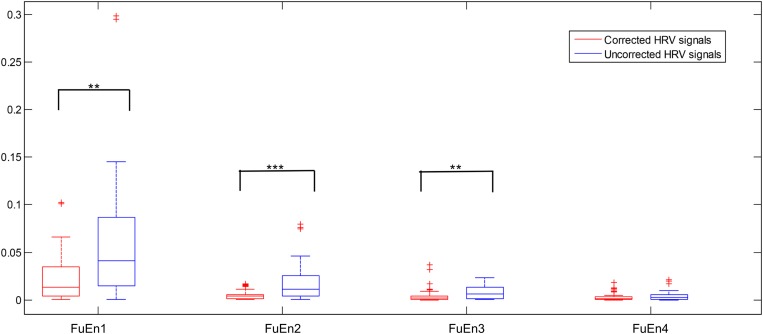
Boxplot of the FuEn indexes computed from the first four IMFs from the 1st 2-min uncorrected and corrected HRV beats, respectively (**P < 0.01 and ***P < 0.001, respectively).

We computed the FuEn indexes of different IMFs obtained from EEMD decomposition for uncorrected and corrected 1st 2-min interval HRV signals, respectively, as shown in Figure 4 (FuEn1, FuEn2, FuEn3, and FuEn4 represent the FuEn features extracted from the first to fourth IMF obtained from EEMD decomposition. This notation also applies to other entropy features such as DisEn, IMPE, RdisEn, and RenEn in the following section. For example, RdisEn3 represents the RdisEn feature extracted from the third IMF obtained from the EEMD decomposition). There were significant differences between FuEn1, FuEn2, and FuEn3 computed from uncorrected and corrected HRV signals, and the mean values of the FuEn indexes obtained from uncorrected HRV signals were higher than these from corrected HRV signals. The reason behind this was that unexpected data points in RR intervals increase the non-stationarities and complexities of signals analyzed, as shown in Figure 2, and thus distort the measurement reliability of entropy indices, which had been proved by previous research (Magagnin et al., 2011). Pre-processing of HRV signals is thus very necessary for the reliability of the scheme proposed in this work.

As far as we know, the performance of RdisEn and IMPE was affected by the selection of parameter q for RdisEn and parameter s for IMPE (Azami and Escudero, 2016; Shi et al., 2019). To find the optimal parameter values for the entropy algorithms for SCD identification, the *p-*values of RdisEn and IMPE with changing parameter q (1.1 to 2 with a step of 0.3) and s (2 to 6 with a step of 1), respectively, for the first four IMFs for the 1st 2-min HRV signals of normal and SCD subjects were computed by using Student *t*-test. It can be observed from Table 2 that the *p*-value of RdisEn from the first IMF was lowest at *q* = 2 and the *p*-value of IMPE from the fourth IMF is lowest at *s* = 2, so we adopted *q* = 2 and *s* = 2 for the RdisEn and IMPE evaluations in the following study.

**TABLE 2:** *p*-values computed from the first four IMFs obtained from the 1st 2-min HRV signals of normal and SCD subjects with varying parameter *q* for RdisEn and parameter *s* for IMPE.

***q***	**1.1**	**1.4**	**1.7**	**2**	**s**	**2**	**3**	**4**	**5**	**6**
RdisEn1	2.7e−5	1.9e−5	1.44e−5	1.08e−5	IMPE1	0.396	0.039	0.019	0.0476	0.145
RdisEn2	0.035	0.0358	0.0359	0.036	IMPE2	2.1e−7	8.08e−4	0.0038	0.3235	0.967
RdisEn3	7.1e−4	7.8e−4	8.03e−4	8.0e−4	IMPE3	1.3e−7	1.4e−7	1.0e−4	0.7536	0.0097
RdisEn4	0.0018	0.0022	0.0026	0.0028	IMPE4	1.4e−4	3.02e−5	9.17e−5	7e−4	0.006

**FIGURE 5:**
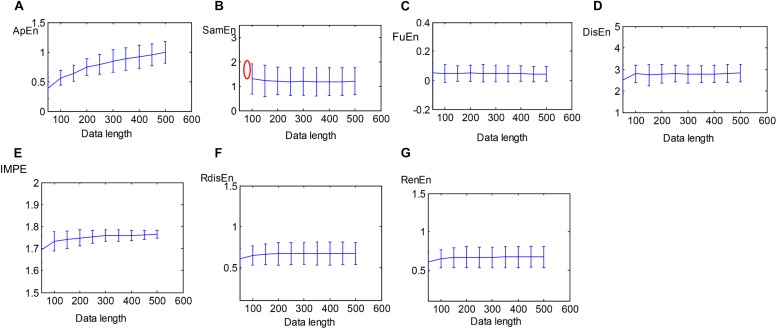
Errorbars of **(A)** ApEn, **(B)** SamEn, **(C)** FuEn, **(D)** DisEn, **(E)** IMPE, **(F)** RdisEn, and **(G)** RenEn computed from SCD HRV signals with varying length (red mark represents undefined value).

A total of 40 HRV signals with varying lengths from 50 to 500 with a step of 50 were extracted from SCD HRV signals to evaluate the sensitivity of the entropy algorithms to the data length. The performances of approximation entropy (ApEn), sample entropy (SamEn) (Porta et al., 2017), and the five entropy indexes aforementioned were assessed as a function of the length of HRV signals, illustrated in Figure 5. The curve of the ApEn value monotonically incremented with data length, and there was an undefined value for SamEn at the data length of 50, as shown in Figures 5A,B, suggesting the instability of the ApEn measurement and failure of the SamEn measurement to quantify the complexity of short-term HRV signals, which was consistent with the previous study (Li et al., 2015). Additionally, another study reported that ApEn easily brought about a biased estimator due to the effect of self-matches when applied to the analysis of a short-term time series (Porta et al., 2007). Figures 5C–G demonstrates that the five entropy indicators used in this work still remain stable at a data length greater than 100 and that there were no undefined values for measuring the irregularity of short-term HRV signals, indicating that the five entropy indicators were insensitive to the data length and suitable for the following analysis of short-term HRV beats.

**FIGURE 6:**
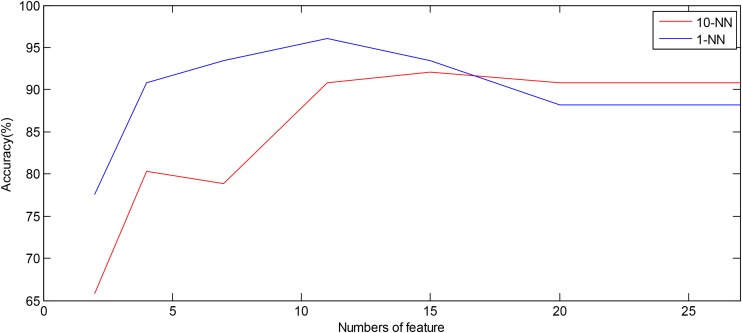
Plot of number of features ranked by the entropy method versus accuracy using the 1-NN and 10-NN classifiers by using combined features, respectively.

Table 3 presents the values (mean and SD) of the 20 EEMD-entropy features acquired from the first 2-min and 5-min intervals of normal and SCD HRV signals. There were noticeable differences among most of the EEMD-based entropy measures for distinguishing between the normal patients and those at risk of SCD for the first 2-min HRV beats. Similar results were obtained by these entropy metrics in assessing disorder of the first 5-min HRV beats of the two groups, implying the reliability of the EEMD-based entropy metrics. For most entropy features, where the performance of the entropy features extracted from the lower IMFs was better, the features with significant difference between normal subjects and SCD patients were mostly extracted from the first third of IMFs, implying that the selection of the first four IMFs for the following analysis was appropriate (Table 3). Seven time/frequency-time features and 20 EEMD-based entropy features were computed from the 1st 2-min HRV beats and ranked by various methods such as *t*-test, entropy, ROC, Wilcoxon, and Bhattacharyya. The ranked features were fed into 1-NN and 10-NN one by one to obtain the highest accuracy. Figure 6 showed classification performances by using the entropy ranking method for 1^st^ 2-min intervals. It is obvious from Figure 4 that, in distinguishing normal from SCD-affected HRV signals, the highest accuracy of 96.1% was achieved using the 1-NN classifier with 11 features. The classification results with the highest accuracy by using the various ranking methods are tabulated in Table 4 for the 1st 2-min. It was not difficult to find that the best classification was achieved by the entropy method, so we adopted the ranking method of entropy in the following six cases for SCD classification. Furthermore, the SCD detection scheme was also implemented by using EEMD-based and classical linear estimators, respectively, for comparison. Obviously, Table 4 shows that the performance of SCD detection by using the EEMD-based entropy of HRV signals was superior to that by using the classical linear method (94.7% vs.86.8%). Furthermore, the performance by using the combination of parameters performed better than the other two methods, suggesting that classical linear domain metrics for SCD identification are an important complement to the non-linear analysis of HRV signals proposed in this paper; this is consistent with previous research (Voss, 1996; Guzzetti et al., 2005).

**TABLE 3:** Twenty EEMD-based entropy features extracted from normal and SCD HRV signals 1st 2-min and 1st 5-min before SCD occurrence.

**Feature**	**1st 2-min**		***P*-Value**	**1st 5-min**		***P*-Value**
	**Normal**	**SCD**		**Normal**	**SCD**	
FuEn1	0.0021 ± 0.0023	0.027 ± 0.025	6.6e−6	0.0023 ± 0.003	0.018 ± 0.023	9.3e−5
FuEn2	0.0018 ± 0.0016	0.0051 ± 0.005	3.35e−4	0.0017 ± 0.004	0.004 ± 0.005	4.15e−4
FuEn3	7.8e−4 ± 5.8e−4	0.0046 ± 0.008	0.005	7.8e−4 ± 6.4e−4	0.0033 ± 0.004	9.3e−4
FuEn4	4.9e−4 ± 7.1e−4	0.0027 ± 0.004	0.002	4.8e−4 ± 5.5e−4	0.0023 ± 0.004	0.003
DisEn1	3.311 ± 0.71	2.793 ± 0.466	2.15e−8	3.33 ± 0.194	2.916 ± 0.419	6.67e−7
DisEn2	3.029 ± 0.15	2.99 ± 0.479	0.6926	3.06 ± 0.217	3.067 ± 0.402	0.913
DisEn3	2.683 ± 0.11	2.61 ± 0.443	0.279	2.711 ± 0.114	2.634 ± 0.353	0.021
DisEn4	2.294 ± 0.13	2.33 ± 0.234	0.403	2.301 ± 0.128	2.336 ± 0.278	0.495
IMPE1	1.742 ± 0.034	1.75 ± 0.0203	0.3961	1.75 ± 0.026	1.762 ± 0.081	0.024
IMPE2	1.708 ± 0.045	1.76 ± 0.0273	2.1e−7	1.73 ± 0.048	1.767 ± 0.018	3.4e−5
IMPE3	1.416 ± 0.084	1.52 ± 0.073	1.3e−7	1.45 ± 0.048	1.523 ± 0.051	4.2e−8
IMPE4	1.089 ± 0.078	1.17 ± 0.109	1.4e−4	1.15 ± 0.053	1.211 ± 0.0822	3.0e−4
RdisE1	0.885 ± 0.051	0.778 ± 0.1275	1.08e−5	0.843 ± 0.065	0.76 ± 0.135	0.003
RdisEn2	0.857 ± 0.04	0.815 ± 0.113	0.036	0.818 ± 0.06	0.795 ± 0.117	0.3
RdisEn3	0.897 ± 0.03	0.83 ± 0.111	8.07e−4	0.848 ± 0.04	0.789 ± 0.111	0.003
RdisEn4	0.916 ± 0.025	0.878 ± 0.073	0.003	0.857 ± 0.043	0.835 ± 0.102	0.238
RenEn1	13.204 ± 1.29	11.92 ± 1.21	2.66e−5	14.95 ± 1.76	13.61 ± 1.442	4.8e−4
RenEn2	10.961 ± 1.46	12.08 ± 1.81	0.004	12.83 ± 1.41	13.74 ± 1.401	0.006
RenEn3	8.948 ± 1.73	9.36 ± 1.57	0.278	11.59 ± 1.49	12.445 ± 1.73	0.025
RenEn 4	5.996 ± 1.31	6.96 ± 1.84	0.011	9.5 ± 1.49	9.88 ± 1.643	0.405

**TABLE 4:** Classification of highest accuracy for the 1st 2-min interval by using various domain features.

**Feature**	**Ranking method**	**Classifier**	**Number of features**	**Accuracy**	**Sensitivity**	**Specificity**
Combined features	Entropy	1-NN	11	96.1%	95%	97.2%
	*t*-test	10-NN	10	92.1%	85%	100%
	ROC	10-NN	10	94.7%	92.5%	97.2%
	Wilcoxon	1-| NN	11	93.47%	92.5%	94.4%
	Bhattacharyya	1-NN	10	90.8%	90%	91.6%
EEMD-based entropy features	*t-t*est	1-NN	10	94.7%	92.5%	97.2%
Time and frequency domain features	*t-t*est	10-NN	7	86.8%	100%	72.2%

**FIGURE 7:**
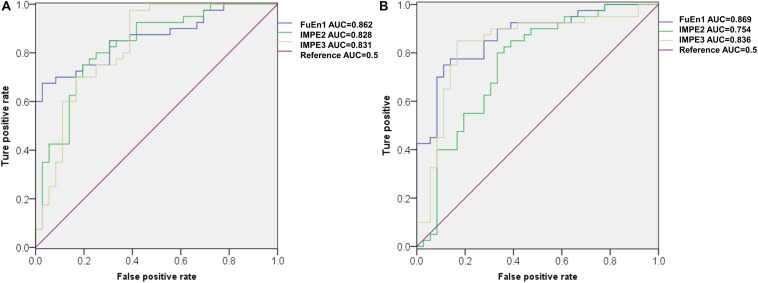
ROC cures of FuEn1, IMPE2, and IMPE3 extracted from **(A)** 1st 2-min and **(B)**1st 5-min HRV signals.

Figure 7A illustrates the ROC curves of three features obtained using ROC analysis for the 1st 2-min, where the AUC values of these features were ranked top three among all the 27 features. Notably, FuEn1, IMPE2, and IMP3, derived from the EEMD-based entropy features, outperformed all classical linear features and showed a superior capability to distinguish normal from SCD HRV signals. To further verify the reliability of these three features, the ROC curves of the three features when computed from the 1st 5-min HRV signals are exhibited in Figure 7B. We can observe that the AUC values of FuEn1 and IMPE3 remain almost constant.

Table 5 showed the maximum accuracies based on the integrated features on all seven cases (i.e., the 1st 2-min, 2nd 2-min, 3rd 2-min, 4th 2-min, 5th 2-min, 6th 2-min, and 7th 2-min) were achieved using different classifiers with different numbers of features. It deserved mentioning that we achieved a higher accuracy of 96.1%, sensitivity of 97.5%, and specificity of 94.4% using the 10-NN classifier with 10 features for SCD detection 14 min before SCD onset.

**TABLE 5:** Maximum accuracy obtained on all seven cases by using combined features.

**Cases (Classifiers)**	**Features**	**Accuracy**	**Sensitivity**	**Specificity**
First two minutes (1-NN)	11	96.1%	95%	97.2%
Second two minutes (10-NN)	11	90.8%	92.5%	88.9%
Third two minutes (10-NN)	10	97.4%	97.5%	94.4%
Fourth two minutes (1-NN)	4	94.7%	95%	94.4%
Fifth two minutes (10-NN)	5	94.7%	95%	94.4%
Sixth two minutes (1-NN)	8	93.4%	95%	91.7%
Seventh two minutes (10-NN)	10	96.1%	97.5%	94.4%
Average		94.7%	95.5%	93.6%

## Discussion

Table 6 clearly shows that two different prediction time resolutions (1-min and 2-min intervals) have been adopted for SCD prediction. As this interval is over 2 min, it would result in too small a prediction resolution and thus will influence the efficiency of the prediction.

**TABLE 6:** Summary of previously reported early SCD detection using ECG/HRV signals.

**Author (year)**	**Data (ECG or HRV)**	**Total no. of features**	**Method (features)**	**Classification**	**Results**
**Early SCD detection using 1-min interval ECG/HRV signals**					
**Ebrahimzadeh and Pooyan (2011)**	35 normal and 35 SCD (HRV) Source: Normal Sinus Rhythm (NSR) database and Sudden Cardiac Death Holter (SCD) database	20	Linear and non-linear methods (time-domain features (5); frequency-domain features (4); time-frequency domain features (11))	KNN, Multilayer perceptron (MLP)	Acc = 91.23% (2nd 1 min before)
**Ebrahimzadeh et al. (2014)**	35 normal and 35 SCD (HRV) Source: NSR database and SCD database	24	Linear and non-linear methods (time-domain features (5); frequency-domain features (4); time-frequency domain features (11); non-linear features (4))	KNN, Multilayer Perceptron Neural Network	Sen = 83.75% Acc = 83.93% (4th 1 min before)
**Acharya et al., 2015a**	36 normal and 40 SCD (ECG) Source: NSR database and SCD database	18	DWT, non-linear methods (non-linear features (6))	SVM, DT, KNN	Sen = 92.50% Spe = 91.67% Acc = 92.11% (4^th^ 1 min before)
**Acharya et al., 2015b**	36 normal and 40 SCD (HRV) Source: NSR database and SCD database	10	Recurrence Quantification Analysis, non-linear methods (RQA parameters (10))	SVM, PNN, KNN, DT	Sen = 85% Spe = 88.8% Acc = 86.8% (4th 1 min before)
**Mirhoseini et al. (2016)**	18 normal and 19 SCD (HRV) Source: NSR database and SCD database	22	Linear and non-linear methods (time-domain features (5); frequency-domain features (4); time-frequency domain features (10); non-linear features (3))	SVM	Spe = 89.5% Acc = 83.24% (1st 1 min before)
**Fujita et al. (2016)**	18 normal and 20 SCD (HRV) Source: NSR database and SCD database	9	Wavelet transform, non-linear methods (non-linear features (9))	DT, SVM, KNN	Sen = 95% Spe = 94.4% Acc = 94.7% (4th 1 min before)
**Ebrahimzadeh et al. (2017)**	35 normal and 35 SCD (HRV) Source: NSR database and SCD database	24	Linear and non-linear methods (time-domain features (5); frequency-domain features (4); time-frequency domain features (11); non-linear features (4))	MLP	Sen = 82.67% Spe = 85.09% Acc = 83.88% (12th 1 min before)
**Ebrahimzadeh et al. (2018a)**	35 normal and 35 SCD (HRV) Source: NSR database and SCD database	24	Linear and non-linear methods (time-domain features (5); frequency-domain features (4); time -frequency domain features (11); non-linear features (4))	MLP, KNN	Acc = 83.96% (4th 1 min before)
**Ebrahimzadeh et al. (2019)**	35 normal and 35 SCD (HRV) Source: NSR database and SCD database	28	Linear and non-linear methods (time-domain features (5); frequency-domain features (4); time-frequency domain features (11); non-linear features (8))	MLP, SVM, KNN	Sen = 85.72% Spe = 82.86% Acc = 84.28% (13th 1 min before)
**Early SCD detection using HRV signals of 2-min interval**					
**Shen et al. (2007)**	20 normal and 23 SCD Source: NSR database and SCD database	4	Non-linear methods (time-frequency domain features (4))	Artificial neural networks (ANN); back propagation (BP)	Acc = 87.5% (1^st^ 2 min before)
**Murukesan et al. (2014)**	18 normal and 20 SCD Source: NSR database and SCD database	34	Linear and non-linear methods, Poincaré plot analysis (time-domain features(15); frequency-domain features(13); non-linear features (6))	SVM, PNN	Sen = 93.33% Spe = 100% Acc = 96.36% (1^st^ 2 min before)
**Current Study**	36 normal and 40 SCD Source: NSR database and SCD database	27	EEMD, linear and non-linear methods (time-domain features(3); frequency-domain features (4); non-linear features (5))	KNN	Sen = 95%; Spe = 97.2% Acc = 96.1% (1^st^ 2 min before) Average acc = 94.7% (14 min before)

*Sen, sensitivity; Spe, specificity; Acc, accuracy.*

In studies on the use of ECG/HRV with a 1-min interval for SCD prediction, Ebrahimzadeh et al. have done much and acquired great achievements (Ebrahimzadeh and Pooyan, 2011; Ebrahimzadeh et al., 2014, 2017, 2018a, 2019; Ebrahimzadeh and Araabi, 2016). They used a total of 20 features extracted from time, frequency, and time-frequency domains and classified the SCD and normal subjects with an accuracy of 99.16 and 91.23% for the first and the second minutes, respectively, prior to SCD onset (Ebrahimzadeh and Pooyan, 2011). The combination of non-linear and time-frequency features coupled with the KNN and MLP classifiers resulted in accuracies of 99.73, 96.52, 90.36, and 83.93% for the first to fourth 1 min before SCD onset (Ebrahimzadeh et al., 2014). In 2017, they proposed a local feature subset selection method to extract the best combination of features from a total of 24 combined features, and therefore the selected features in each 1-min HRV interval were different. The proposed method had the ability to predict SCD 12 min before occurrence and reported 82.67% sensitivity, 85.09% specificity, and 83.88% accuracy for the 12th 1-min. The experimental results in the study indicated that time-frequency and non-linear features performed better in separating normal from high-risk SCD HRV signals compared to classical features including time and frequency features. Recently, in 2019, they introduced a combined model with a local feature subset selection method and the Mixture of Expert (ME) classifier, which can predict SCD 13 min prior to the onset with 84.24% sensitivity, 85.71% specificity, and 82.85% accuracy. Several automated SCD detection models were introduced by Acharya et al. (2015a, b), Fujita et al. (2016). Based on DWT and non-linear features, namely Detrended Fluctuation Analysis, Fractal Dimension, Hurst’s exponent, ApEn, SampEn, and Correlation Dimension, an automated SCD detection scheme was designed to differentiate normal and pre-SCD events by using ECG signals and achieved 92.50% sensitivity, 91.67% specificity, and 92.11% accuracy for the 4th minute before the onset of SCD (Acharya et al., 2015a). Fujita et al. introduced a novel SCD prediction algorithm by using non-linear features (RenEn, FuEn, Tsallis entropy, Hjorth’s parameters and energy of DWT coefficients) and were capable of discriminating a person at risk of SCD from normal subjects with 94.7% accuracy for the 4th minute prior (Fujita et al., 2016).

In a study on HRV in 2-min intervals for SCD prediction, Shen et al. (2007) applied fast Fourier transforms to 2-min duration HRV signals before the onset of SCD and then extracted frequency domain features from the corresponding standard segments. The proposed method achieved an accuracy of 67.44% for distinguishing SCD risk groups from normal groups. Murukesan et al. performed SCD prediction 2 min before the incident with the help of a total of 34 features, including 13 frequency domains, 15 time domains, and 6 non-linear domains, and obtained accuracies of 96.36 and 93.64% for SVM and PNN, respectively (Murukesan et al., 2014).

From Table 6, it is evident that the prediction time of most studies is 4 min at the most. The clinician has insufficient time to provide timely and efficient therapy for patients at risk of SCD outside a hospital. The prediction time of individual studies is extended from 4 to 13 min, but using more a short-term interval (1-min) may result in unreliability of frequency domain and entropy features, so the recommended duration of short-term recording is 2 to 5 min (Malik, 1996). Synthesizing the prediction resolution and reliability of the SCD detection scheme, we selected HRV signals with a 2-min interval for analysis.

It has been reported that SCD is a fatal cardiovascular disease that can involve abnormality of the autonomic nervous system (ANS), and frequency domain analysis of HRV signals is widely employed to evaluate the activity of ANS for SCD risk stratification (Wellens et al., 2014; Malik, 1996). In this paper, we investigated the performance of EEMD-based entropy indexes extracted from HRV beats on SCD identification for the following reasons. Firstly, the superiority of the EEMD method is that it decomposes time series into IMFs in a data-dependent and adaptive manner, making it suitable for the analysis of unstable and non-linear HRV signals (Sharma et al., 2015), unlike the DWT method, in which decomposition is related to a predetermined wavelet basis function. Secondly, IMFs obtained by EEMD are representative of the intrinsic oscillatory and frequency modes; the fast oscillation modes are contained by the lower-order IMFs, and the slow oscillation modes are captured by the higher-order IMFs, that is to say, the lower IMFs contain more energy. We therefore selected the first four IMFs obtained by EEMD for HRV analysis since the first four IMFs occupied almost all the energy of signals analyzed, as shown in Figure 3. Studies have reported that the frequency components of IMFs are arranged in descending order: the lower IMFs capture higher-frequency components and vice versa (Sharma et al., 2015). Moreover, entropy, a powerful tool for quantifying the disorder and irregularity of dynamic systems, has been widely used for HRV signals recently, as tabulated in Table 6. Therefore, EEMD-based entropy HRV signals analysis provides a new way of assessing the complexity of the rhythm variation of ANS so as to unearth significant clinical information related to diseases. Li et al. (2019) proposed a novel descriptor, namely sliding trend fuzzy approximate entropy (SITr-fApEn), based on the empirical mode decomposition (EMD) method for analyzing ANS with obstructive sleep apnea (Li et al., 2019), and Pan et al. (2019) introduced a multi-frequency components entropy (MFC-En) based on EMD for CHF classification. MFC-En was verified to be a useful tool for CHF measurement by evaluating the irregularity of rhythm variations of the ANS.

In this paper, considering that there were undefined values or computation instability as some entropy measures were performed on short-term series, we first tested the reliability of the ApEn, SamEn, RenEn, FuEn, DisEn, RdisEn, and IMPE measures for short-term time series. Figure 5 shows that the RenEn, FuEn, DisEn, RdisEn, and IMPE measures performed stably in contrast to the ApEn and SamEn measures; therefore, the RenEn, FuEn, DisEn, RdisEn, and IMPE measures were adopted in the subsequent analysis. Table 3 illustrates that most of the EEMD-based entropy measures computed from IMFs could significantly distinguish patients affected by SCD from normal subjects on the basis of 2-min interval HRV beats. The FuEn1, IMPE2, and IMPE3 measures, among all of the HRV measures including seven time-frequency and 20 EEMD-based entropy indexes, achieved the top three AUC values of 0.862, 0.828, and 0.831, respectively, for 2-min HRV beats, and the mean values of the three entropy metrics in patients affected by SCD were higher than those in normal subjects, as shown in Figure 7A and Table 3, suggesting that SCD patients had more disorder of ANS than normal subjects. In a study on HRV analysis, 5-min RR intervals were considered to be more suitable for autonomic nerve assessment (Li et al., 2019). We also assessed the performances of the EEMD-based entropy metrics on 5-min HRV beats, and the simulation results showed that these EEMD-based entropy metrics achieved comparable performance (Table 3 and Figure 7B), further suggesting that the FuEn1 and IMPE3 measures can be used as novel descriptors for quantifying disorder of ANS affected by SCD.

Classical time and frequency indexes such as RMSSD, SDNN, PNN50, VLF, LF, HF, and LF/HF were used for SCD detection in this paper. These indexes have been proved to be useful tools by previous studies (Voss, 1996; Guzzetti et al., 2005; Sammito and Bockelmann, 2016). In this paper, we achieved an accuracy of 94.7 and 86.8% by using EEMD-based entropy and classical linear methods, respectively, for 1st 2-min HRV beats, implying the superiority of the EEMD-based entropy methods proposed for SCD detection (Table 4). Subsequently, a novel SCD detection technique was developed based on classical linear and EEMD-based entropy methods to analyze 14-min HRV signals and achieved an average sensitivity of 93.6%, specificity of 95.5%, and accuracy of 94.7% (Table 4). The novelty of our proposed methods was that the prediction time was firstly extended from 4 min to 14 min by analyzing 2-min interval HRV signals with a high average accuracy, and the FuEn1, IMPE2, and IMPE3 indexes from the EEMD-based entropy indexes were proved to be powerful descriptors for measuring the complexity of ANS in SCD patients (Table 3 and Figure 7). Additionally, a 10-fold cross-validation algorithm made our proposed system more robust and reliable.

This work has some limitations, in that important confounding factors such as age, sex, and pathological condition were not taken into account because of the small amount of data available for SCD detection. There is therefore a great necessity that the proposed SCD detection algorithm be applied to a large data set before its implementation for clinical purposes. Secondly, some useful methods such as symbolic dynamics, renormalized entropy (Voss, 1996) and conditional entropy (Porta et al., 2001, 2017) for HRV signals analysis were not used in this paper. In the future, we will consider these limitations to improve the SCD detection scheme proposed.

## Conclusion

In the area of early SCD detection, providing clinical early warning information is the biggest challenge in cardiology. In this work, we have proposed novel algorithms based on classical linear and EEMD-based entropy methods, which have the capacity to predict SCD occurrence up to 14 min prior, with an average accuracy of 94.7%, sensitivity of 95.5%, and specificity of 93.6%. Moreover, the EEMD-based entropy estimators proposed in this paper showed significant differences between SCD patients and normal individuals. The results also showed that the EEMD-based FuEn1 and IMPE3 indexes were particularly suitable measurements for SCD identification, and these indexes, as novel indices, can be used to quantify the complexity of the rhythm variations of the autonomic nervous system when affected by SCD.

## Data Availability Statement

Publicly available data sets were analyzed in this study. The data sets used are from the MITBIH Database available online at https://archive.physionet.org/physiobank/database/sddb/; https://archive.physionet.org/physiobank/database/nsrdb/.

## Author Contributions

MS and HH contributed the majority of the writing and conducted major parts of the experiments. WG, RW, CZ, YJ, FZ, and SR conducted some experiments and contributed to the methodology. BS supervised the work and revised the manuscript.

## Conflict of Interest

The authors declare that the research was conducted in the absence of any commercial or financial relationships that could be construed as a potential conflict of interest.
